# Supplementary material for: A cross-sectional survey on the effectiveness of public health campaigns for changing knowledge, attitudes, and practices in Kenyan informal settlements during the COVID-19 pandemic
Source: PLoS One. 2023 Dec 22;18(12):e0294202. doi: 10.1371/journal.pone.0294202 (PMC10745220; doi:10.1371/journal.pone.0294202)
Supplement: S1 File — (PDF) [file pone.0294202.s001.pdf]

Final V4- 2020-07 ADRA-UoM-Assessing the effectiveness of bespoke locally sourced face masks in reducing COVID-19 transmission within informal settlements in Kenya

Geo-Location

Please take the geolocation of the Respondent before getting into the house or any other suitable place for the interview.

latitude (x.y °)

longitude (x.y °)

altitude (m)

accuracy (m)

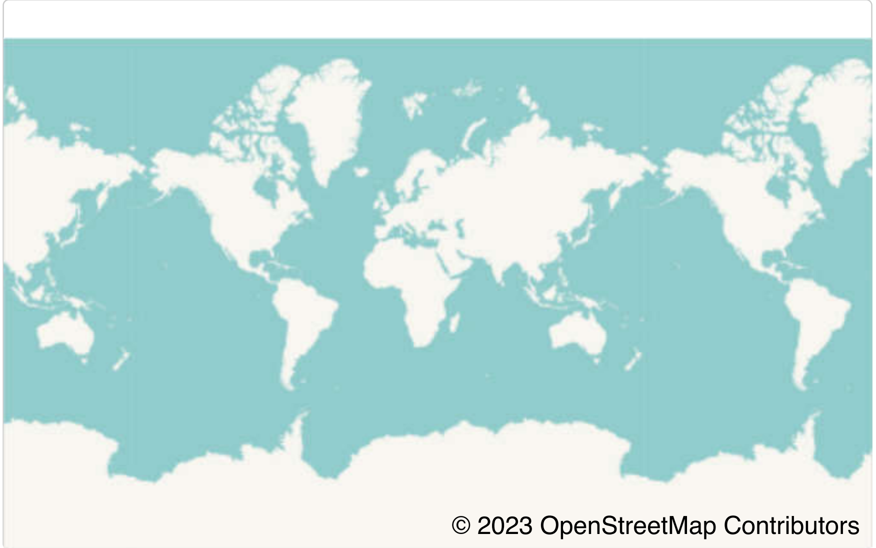

Name of Informal Settlement

- ◯ Kibera, Nairobi
- ◯ Rodah, Nakuru
- ◯ Daraja/Nubian, Kisii

Enumerator's Name

Please write your Name or Initials here (Example: James Martin or JM). Try to maintain Consistency for ease of tracking

Introduce Yourself, The name of the organization you represent. The goal of the survey

Respondent Consent

You are being invited to take part in a research study aimed at evaluating the use of face masks at a time of a major epidemic in Kenya. Before you decide whether to take part, it is important for you to understand why the research is being conducted and what it will involve. Please take time to read the information leaflet we have provided carefully before deciding whether to take part and discuss it with others if you wish. Please ask if there is anything that is not clear or if you would like more information. Thank you for taking the time to read it. Do you wish to continue?

- ◉ Yes
- ◯ No

Consent Form (Scan)

Take a picture of the signed consent form

Click here to upload file. (< 5MB)

1. Name of Respondent

Respondent's Gender/Sex

- ◯ Male
- ◯ Female
- ◯ Other

2.Age of Respondent

---

3. Address of Responden:

4.Sample ID

5.Highest attended Educational Level (Please choose one of the following responses)

- ◯ Never attended education
- ◯ Primary school
- ◯ Secondary school
- ◯ College
- ◯ University degree
- ◯ Postgraduate degree

6.In a typical week, how many days do you work?

---

7. Distance from Home to workplace

Approximately how far away from your home (in km) is your place of work?

8a. Time spent at home on working days

In a typical working day, approximately, how many hours do you spend: a) In your home?

____

8b. Time spent In others' homes on working days

In a typical working day, approximately, how many hours do you spend: b) In other people's homes?

____

8c. Time spent at work on working days

In a typical working day, approximately, how many hours do you spend: c) At work?

____

9a. Time spent at home on non-working days

During days when you do not work, approximately how many hours do you spend: a) In your home?

____

9b. Time spent In others' homes on non-working days

During days when you do not work, approximately how many hours do you spend: b) In other people's homes?

____

Sketch Map of respondent's neighbourhood (Scan)

Please let the respondent sketch their neighbourhood on the map provided (take a picture of the sketch map) also submit the original hard copy of the sketch

Click here to upload file. (< 5MB)

Sketch Map of respondent's movement (Scan)

Please let the respondent sketch on the map provided how far they may go outside the neighbourhood (take a picture of the sketch map) also submit the original hard copy of the sketch

Click here to upload file. (< 5MB)

10a.Time spent in the neighbourhood on working days

During a typical working day, approximately how many hours do you spend: Within your neighbourhood (as defined on the map)

____

10b.Time spent outside the neighbourhood on working days (but within settlement)

During a typical working day, approximately how many hours do you spend: Outside your neighbourhood, but within the settlement?

____

10c.Time spent outside the settlement on working days

During a typical working day, approximately how many hours do you spend: Outside the settlement?

____

11a.Time spent in the neighbourhood on non-working days

During days when you do not work, approximately how many hours do you spend: Within your neighbourhood only (as defined on the map)?

____

11b.Time spent outside the neighbourhood on non-working days ? (but within settlement)

During days when you do not work, approximately how many hours do you spend: Outside your neighbourhood, but within the settlement?

____

11c.Time spent outside the settlement on non-working days

During days when you do not work, approximately how many hours do you spend: Outside the settlement?

____

12.Number of daily contacts on a typical week-day

On an average week-day how many people (other than your immediate households) will you come into close contact with (come within 2 meters for over 2 minutes)

- ○ <5
- ○ 5-10
- ○ 10-15
- ○ 15-20
- ○ >25

13. Number of daily contacts on a non-working day

On a typical day when you do not work (e.g. week end or holiday day etc), how many people (other than your immediate households) will you come into close contact with (come within 2 meters for over 2 minutes)

- ○ <5
- ○ 5-10
- ○ 10-15
- ○ 15-20
- ○ >25

14. Do you currently use a face mask to protect yourself from Covid 19 (please choose a response):

- ○ Yes
- ○ No

15. If NO please say why you do not use face masks:

- [ ] It's too expensive
- [ ] It's uncomfortable to use
- [ ] It's not effective
- [ ] It's cumbersome and unattractive to use
- [ ] All or some of the above

16. If YES How frequently do you use the mask?

- ○ Always when I go out
- ○ Most of the time but not always when I go out
- ○ Sometimes when I go out
- ○ Never

17. Where did you get the mask from?

- [ ] Provided by government or an NGO
- [ ] Bought from internet
- [ ] Bought from shop
- [ ] Made it myself/family member

18.How much did you spend on purchasing your mask or purchasing material for making your mask ? (Local Currency KES)

____

19. Do you expect a person with chronic cough to use a face mask?

- ○ Yes
- ○ No
- ○ Uncertain

20. When you wear your mask what part of the face do you cover

- ○ Mouth only
- ○ Nose only
- ○ Mouth and nose
- ○ Varies

21. Is you mask a reusable mask or disposable mask or both?

- [ ] reusable mask
- [ ] disposable
- [ ] Sometimes Reusable and sometimes disposable

22. If reusable, how do you maintain your mask (Please describe)

- ○ Washing
- ○ dry cleaning
- ○ dipping in anti-septics
- ○ none of the above

23. If reusable, how frequently do you wash your mask?

- ○ Washing
- ○ dry cleaning
- ○ dipping in anti-septics
- ○ none of the above

24 a .If reusable, how frequently do you replace your mask

- ○ Daily
- ○ 2-3 days
- ○ Weekly
- ○ Fortnightly
- ○ Monthly
- ○ Never

24 b If disposable, how do you dispose the used mask?

____

25. How frequently do you wash your hands before wearing the mask?

- ○ Always
- ○ most of the time
- ○ Sometimes
- ○ Never

26. How frequently do you handle or reposition the mask when you are wearing one?

- Very frequently
- Frequently
- Infrequently
- Never

27. Have you had any information or training provided on the correct use of masks

- None
- Some information but not adequate
- Adequate information
- N/A

28.Do you believe wearing a mask is useful to prevent the spread of Corona virus in your community

- Yes definitely
- Possible but not certain
- Do not know

29. If it is proven that wearing of a mask will help prevent the spread, will you wear a mask?

- Definitely yes
- Possibly yes
- Possibly No
- Definitely No

30. Can you suggest some reasons as to why people may not wish to wear a mask during the Covid 19 epidemic

____

31. Do you feel that use of mask can be harmful at a time of a respiratory pandemic

- Yes
- No
- Uncertain

32.If you think the use of a mask can be harmful can you tell us why you think so

- [ ] They affect breathing
- [ ] They can be a medium for bacterial growth
- [ ] People may get a false sense of security and violate other safety rules
- [ ] They make people sick
- [ ] Don't know

Addtitional Questions

33.What do you understand about Social distancing?

34. How many metres should we keep from one another?

- 1.5 metres
- I metre
- 2 metres
- Don't know

35. Are people in your area able to maintain social distance?

- Yes
- No

36. What are some of the reasons why people in your area not able to keep social distance?

37.How often do you wash your hands?

- less than 3times
- 4-5 times
- As often as I see water
- Depending on what I have touched
- when I come from outside

38.What are the challenges with hand washing?

- lack of water
- lack of soap
- forgetfulness
- fatigue

39. How long should we wash our hands?

- 10 minutes
- 5 minutes
- 20 seconds
- 2 minutes

40.How do you perceive government directives on Covid 19?

- [ ] Believe them
- [ ] mistrust
- [ ] Obedience
- [ ] Useless

41. Have you been tested for COVID-19?

- ◯ Yes
- ◯ No

42. What are the reasons you did not take the test?

- ◯ Fear of quarantine
- ◯ Fear of testing positive
- ◯ Stigma
- ◯ Not available
- ◯ Not sick

43. Would you be willing to undergo a Covid 19 test if available?

- ◯ Yes
- ◯ No

44. Do you know anyone who has been tested for Covid 19?

- ◯ Yes
- ◯ No

45 a. Are you aware that the government had restricted travelling to and from some counties

- ◯ Yes
- ◯ No
- ◯ Don't know

46b. If Yes why do you think the restricted the movement

- ◯ To stop spread of COVID-19
- ◯ For quarantine
- ◯ Don't know

47. Do you know anyone who has been in quarantine?

- Yes
- No

48. If yes, what experiences did they have of the quarantine facility?

---

49. How long was the quarantine period?

- one week
- two weeks
- 14 days
- 21 days
- Don't know

50. One of the measures the Ministry of health has developed to curb the spread of Covid -19 is isolation. What are your thoughts about this measure?

---

51. What challenges do you think people in your community would experience if one of you is asked to self-isolate?

Thank you

Thank the participant for accepting to take part in the survey and/or for their time (even if consent declined). Remember and remind the participants to stay safe and observe all the health measure put in place by government for their own personal safety..

Enumerator's Notes/Comments

Write any information/observations and notes of that you feel may be relevant to the study but was not captured in any on the question above (you can also send a scan of your field notes.)
