# Supplementary material for: A cross-sectional survey on the effectiveness of public health campaigns for changing knowledge, attitudes, and practices in Kenyan informal settlements during the COVID-19 pandemic
Source: PLoS One. 2023 Dec 22;18(12):e0294202. doi: 10.1371/journal.pone.0294202 (PMC10745220; doi:10.1371/journal.pone.0294202)
Supplement: S2 File — (DOCX) [file pone.0294202.s002.docx]

*Assessing the effectiveness of bespoke face masks in reducing COVID 19 transmission within informal settlements in Kenya*

*************

**Health Promotion Campaign Detailed Plan**

**Overall Health Promotion Campaign Strategy**

**Community Engagement Meetings**

The inception meetings are intended to promote buy in of the community and its leadership and participate in planning and implementation processes. During the engagement meetings, planning for health promotion will also be prioritized and if possible identify some influential leaders to support health promotions. The meetings to take place between 18^th^ August and 4th of September 2020

**Kibera:**

A total of 9 engagement and planning meetings to be held involving:

1. Deputy County Commissioner,
2. Sub-county public health office
3. Sub-county and ward administrators,
4. Chiefs and village elders and *Nyumba-Kumi*,
5. Local Youth groups and association,
6. Football clubs’ couches,
7. commuter vehicles SACCOS,
8. Religious leaders.
9. Community health volunteers

All planning meetings to involve the health promotion officer and public health officer in charge of Kibera informal settlement Kibera (covering Laini Saba, Makina/DC and Sarangombe Locations). The planning meeting with chiefs and their elders/Nyumba Kumi leaders will be for specific locations/wards. Planning meetings with religious leaders, Matatu SACCOS, Football couches and youth representatives will be for the entire Kibera.

**Nakuru:**

A total of 6 number of engagement meetings to be held involving:

1. Local chiefs, village elders, *nyumba kumi*
2. Public health officers and community health volunteers
3. Women group leaders’ representatives,
4. Youth group leaders’ representatives,
5. Persons with disabilities (group leaders),
6. Religious leaders across Rhoda area.

**Kisii:**

10 engagement meetings to be held with:

1. Minister for Health Kisii County,
2. Director of health Kisii County,
3. County health promotion officer,
4. Infections prevention and Control officer Kisii County,
5. Heads of County health facilities,
6. Chiefs, assistant chiefs,
7. Village elders,
8. Religious leaders,
9. community health volunteers,
10. heads of selected groups of people.

**Actual Health Promotion Campaign**

Daring the entire health promotion and campaign period, the target community are provided with information, explanations and demonstrations on the correct use of masks and the dangers associated with improper use.

This health promotion campaigns are also to include information on regular hand washing with soap as well as other personal hygiene practices depending on local circumstances and locally available resources. **As indicated in the overall project concept, the current research grant does not cover infrastructural costs – such as establishing water supply but will include information sharing on all the aspects.**

1. ***Community sensitization meetings (plot to plot)-*** *1 September to 30th of November 2020*

Community sensitization meetings to be organized by community Health volunteers are part of the community health service delivery mechanism within the counties. (enjoy the level of community acceptability and legitimacy).

ADRA already identified and trained 15 of these volunteers (5 in each settlement) to support the exercise. The volunteers are to work closely with County Public Health Officers and ADRA Filed officers so as to ensure the validity of the messaging.

**Kibera:**

The community meetings will be done with a maximus of 15 people per meeting. A minimum of 3 meetings will be conducted per volunteers totaling to 15 meetings per day.

The 13 villages within Kibera informal settlement has been **zoned into 5 blocks** to make it easier for the five volunteers leading the health campaign activities.

1. Silanga/Undugu/Lindi,
2. Laini Saba/Mashimoni,
3. Darajani/Makina/Karanja,
4. Katwekera/Soweto, and
5. Kianda/Bombolulu/Raila/Olympic.

While each volunteer is responsible for a zone, the volunteers will be working as a team covering a segment of one zone per day. Hence, in every week of 5 days every zone is visited and a segment of the community reached with the health promotion messaging. This is mainly to overcome security challenges observed during the baseline.

During the health promotions, a total of 5 influential leaders will be involved every week. The influential leaders include the chief, assistant chief, village elder, Nyumba Kumi chair person, community health volunteers’ chairman, public health or health promotions.

The health promotional leaflets are to be distributed during the health promotion’s exercise. Each and every plot will be given leaflets for each person after having been engaged in health promotion.

**Nakuru:**

This strategy will involve the volunteers going around the villages of Rhoda informal settlement from plot to plot sensitizing the people on the intended information. They will work closely with the village elders of the respective villages in going around the plots on a daily basis. The public health officer will also be doing random follow up to ensure everything is run according to our set plans and goals and according to the government guidelines. The targeted number of people to be reached will vary daily but the strategy will reach a significant population in the villages in an effective way.

**Kisii:**

Village based health promotions will be done with 5 community health volunteers. Each volunteer will conduct village level health promotion meetings for 5 days in a week. On each day, a volunteer will handle 3 meetings for about 1 to 1.5 hrs. The number of attendants will be 15 (based on government regulations).

The target project area in Kisii has about 55 villages. These villages are further divided into smaller units (clans) ranging from 2 to about 10 for each village. Each volunteer will be handling 11 villages. In a typical day, a volunteer will visit a village and do the health promotions in at least 3 clan units.

Clan elders will be instrumental during the planning, mobilization and health promotional activities (planning for venues, offering security, compliance with COVID 19 protocols, using identification criteria to help select beneficiaries of the mask distribution).

1. ***Use of Worship Place sensitization/Religious leaders meeting-*** *1 September to 30th of November 2020*

The involvement of religious leaders is aimed at tapping on their influence at places of worship to reach to their congregants.

**Kibera:**

One training/sensitization meeting involving religious leaders from 6 churches and 2 mosques. These leaders will in turn be engaged in reaching out to their congregants with key messages on mask usage and its importance as well as other personal hygiene practices including regular hand washing with soap. There will be 2 monthly review meetings with the religious leaders for updates and reporting of success and challenges experienced. Occasionally public health and health promotions officer will be engaged to reach out to churches with bigger populations. The religious leaders’ representatives from 8 places of worship will be engaged in the distribution of leaflets in their respective churches and mosques.

**Nakuru:**

This strategy is set to meet and pass the message to resident meeting in places of worship in Rhoda area. This strategy will be effective to a bigger extent especially after doing an engagement meeting with representatives of worship leaders at Rhoda area prior this exercise. 3 major places of worship will be targeted in each village during the sensitization each weekend. This is expected to help reach out to between 400-500 people every week with key messages on effective use of masks and other health safety practices.

**Kisii**

During the village-based sensitization exercise, some of the community members may miss out on the meetings due to their personal engagements. Such members will be targeted through their places of worship. As a result, 15 places of worship will be used to carry out sensitization. 1 training/sensitization meeting will be held with the religious leaders to equip them with promotional messages to share with their congregations. Each of the religious leaders will be encouraged to share the information during every worship session.

A progress report detailing this sensitization activity will be submitted

Public Health officers will conduct awareness campaigns in few selected places of worship

1. ***Use of Brochure’s Leaflets and flyers***

ADRA Kenya will reproduce/print leaflet/fliers for promotional work. These will be materials already approved and being used by Ministry of Health (the materials have been developed based on WHO guidelines and contextualized for use in Kenya and approved by the government of Kenya). A total of 15,000 leaflets will be produced and distributed.

**Kibera:**

A total of 5,000 leaflets to be distributed across Kibera informal settlement. The distribution will be done using different avenues including churches and mosques which will receive a total of 1500, plot to plot promotions will utilize 2500 leaflets and Roadshows/walk shows will utilize a total of 1000.

**Nakuru:**

A total of 5,000 leaflets to be distributed across Rhoda informal settlement. The distribution will be done using different avenues including churches and mosques which will receive a total of 1500, plot to plot promotions will utilize 2500 leaflets and Roadshows shows will utilize a total of 1000. The leaflets will complement all the other approaches.

**Kisii:**

The brochures and flyers will be used to complement health promotional activities in community forums, churches and health facilities. The distributions will be as follows; churches 2000, community promotions 1700, health facilities 1000, roadshows 300.

1. ***Use of mobile unit motor or people roadshows*** *21st -25th Sep, 8th-14th Oct, 5th-14th November 2020*

Road shows and/or Walk show is aimed at increasing awareness along major roads and the reach people who would not have been reached through plot to plot but are available along the major roads.

**Kibera:**

Walk show will be adopted as an approach in which a group walk-shows an approach where a group of branded influential individuals take to major roads within to engage community members one on one on the use and importance of putting on face masks and demystify the myths around covid-19 which makes people ignore measures placed by the government. A walk show will be done per month for three months. This walk show will incorporate a total of 8-10 influential leaders including the health promotion and public health officers.

**Nakuru:**

This strategy is going to be employed to complement the main strategy of community gatherings and its surely going to reach almost the entire settlement. The messages on health promotion will be conveyed by a trained individual on automobiles fitted with a public address system going around the settlement providing health promotion message. The will be 2 roadshows in a month and 6 roadshows in the 3 months.

**Kisii:**

This strategy will take advantage of the market days in the region. It will be instrumental in reaching out to people who do not attend church or are hardly found in the community. It will be conducted after every 2 weeks on market days at Daraja Mbili and other identified areas within Kitutu Chache South and Kisii Central. During the roadshows, few brochure’s and flyers will be distributed to avoid littering.

1. ***Community/Group Meetings***

**Nakuru:**

There will be 2 meetings each week during weekdays. One meeting will is expected to attract between 50-100 residents of a particular village. Rhonda area is divided into 7 villages and leading to having 3 to 4 group meetings in each village depending with village population. The volunteers and village elders will be used to sensitize people to attend such meetings.

1. ***Use of Social Media***

**Nakuru:**

This is also a complementary strategy that will boost our main plans of health campaign and promotion. This will involve having the softcopy information sent to people social media platforms like whatsapps and facebook etc, and they will share to the local groups of Rhoda residents. This will enhance the campaign especially to the people of age brackets 18-45 yrs.

**Identification of the beneficiaries of masks and Distribution of masks-**50k+ Masks to be distributed

Beneficiaries identification will be an ongoing process during the plot to plot, religious meetings engagements and community engagements. The team will apply a mixed approach to identify beneficiaries of face masks which results to beneficiaries being drawn from different groups ranging from religious leaders to community leaders.

**Kibera:**

The religious leaders, Matatu SACCOs officials, Football couches, Community leaders will help in the identification of beneficiaries from their areas of influence. For instance, religious leaders help in identification of beneficiaries from churches or mosques, football couches from football teams, matatu SACCO leaders from drivers and conductors and community leaders from villages.

The will be planning meeting for the distribution which will be held to involve public health, health promotion, administrative representatives and volunteers. The plan will guide the distribution of masks in different parts of Kibera informal settlement.

Distribution will involve the office of deputy county commissioners, public health, volunteers and religious leaders.

**Nakuru:**

The identification of mask beneficiaries will be done through a meeting with all leaders and stakeholders including representatives of the village elders, nyumba kumi, the disabled, the administrative leaders, the religious leaders, women and youth groups and any other vulnerable groups e.g the HIV/AIDS groups. This will help us to come up with a non-bias and effective criteria on how to go about the whole exercise of masks distribution in the whole Rhoda settlement.

**Kisii:**

ADRA Kenya field worker, Kisii County Director of Health, County health Promotion officer and the Kisii county Infection Prevention and Control officer will come up with the selection criteria. This criterion will be shared with the chiefs, assistant chiefs and village elders to help the volunteers identify such persons in the community. Each of the persons mentioned above will be given a number of people to identify. After identification, there will be a verification of persons identified during awareness meetings.

This distribution will involve personnel from the county government specifically the director of health/ area MCA

**Monitoring, Evaluation, Accountability and Learning (Quality Assurance)**

Monitoring will be done by different people and at different level. Internal monitoring will be done by the field workers during health promotion at community level. Reporting will be real-time sent on daily basis electronically by the volunteers for each event conducted.

**Kibera:**

Monitoring of health promotion will be done on for the first 5 days on a daily basis. Thereafter it will be done on a weekly basis inform of 3 review meetings per months for 3 months. There will be 2 review meetings with religious leaders, 8 review meetings with volunteers on the progress of health promotion. There will also be 1 review meeting with the mask distribution committee which will be formed just before mask are distributed.

**Nakuru:**

Monitoring of health promotion will be done on for the first 5 days on a daily basis. Thereafter it will be done on a weekly basis inform of 3 review meetings per months for 3 months. There will be 2 review meetings with religious leaders, 8 review meetings with volunteers on the progress of health promotion. There will also be 1 review meeting with the mask distribution committee which will be formed just before mask are distributed.

**Kisii:**

The internal monitoring mechanisms will involve the field worker random spot checks. This will be done in collaboration with public health officers, county health promotion officer and infection prevention and control officer. Brief review meetings will be conducted; Monday (plans for the week) and Friday (Recap of weekly activities).

Review meetings with religious leaders and clan elders will be conducted twice for the whole period.

ADRA Program management team will on a daily-basis monitor and review the daily reports submitted by the field teams. ADRA program team will also conduct periodic follow-up and field visits for purposes of quality assurance and compliance.

The independent QA and project oversight team will also conduct periodic reviews and spot checks on the ongoing health promotion activities and conduct periodic field visits where necessary and as will be allowed by the available budget.
