# Supplementary material for: A cross-sectional survey on the effectiveness of public health campaigns for changing knowledge, attitudes, and practices in Kenyan informal settlements during the COVID-19 pandemic
Source: PLoS One. 2023 Dec 22;18(12):e0294202. doi: 10.1371/journal.pone.0294202 (PMC10745220; doi:10.1371/journal.pone.0294202)
Supplement: S3 File — (DOCX) [file pone.0294202.s003.docx]

**Supporting Materials:**

**Supporting Table 1. Individual settlement data related to participant demographics**

|  | Kisii | | | Nairobi | | | Nakuru | | |
| --- | --- | --- | --- | --- | --- | --- | --- | --- | --- |
|  | S1 %  (n=230) | S2 %  (n=231) | *p*-value | S1 %  (n=228) | S2 %  (n=233) | *p*-value | S1 %  (n=233) | S2 %  (n=219) | *p*-value |
| Settlement population | | | | | | | | | |
| Percent | 49.9 | 50.1 | --- | 49.5 | 50.5 | --- | 51.5 | 48.5 | --- |
| Age | | | | | | | | | |
| Years (SD) | 41.0  (15.0) | 39.3 (13.7) | 0.186 | 37.6  (13.4) | 36.3  (12.8) | 0.311 | 41.1 (15.9) | 37.6 (13.6) | 0.006* |
| Gender | | | | | | | | | |
| Female | 50.9 | 49.4 | 0.584 | 50.0 | 51.1 | 0.818 | 51.9 | 49.8 | 0.646 |
| Male | 49.1 | 50.2 |  | 50.0 | 48.9 |  | 48.1 | 50.2 |  |
| Other | 0.0 | 0.4 |  | 0.0 | 0.0 |  | 0.0 | 0.0 |  |
| Highest educational level | | | | | | | | | |
| No education | 8.7 | 6.5 | 0.029* | 1.3 | 2.6 | 0.110 | 4.7 | 4.6 | 0.522 |
| Primary school | 25.2 | 19.5 |  | 34.2 | 24.9 |  | 35.2 | 27.9 |  |
| Secondary school | 35.7 | 46.8 |  | 41.7 | 40.3 |  | 43.8 | 45.2 |  |
| College | 17.0 | 20.3 |  | 16.2 | 25.3 |  | 12.0 | 16.9 |  |
| University | 11.7 | 5.2 |  | 6.1 | 6.4 |  | 3.9 | 5.0 |  |
| Postgrad. degree | 1.7 | 1.7 |  | 0.4 | 0.4 |  | 0.4 | 0.5 |  |

**Supporting Table 1:** Individual settlement data related to participant demographics. Values are shown as percentages %, unless otherwise stated. SD: standard deviation. The total sample size for each settlement in their respective pre-and-post intervention cohort is shown as (n = x) adjacent to the relevant title. S1: Survey 1; S2: Survey 2.

*Significant at 5%

**Supporting Table 2a. Individual settlement data to behaviours around mask use and procurement**

|  | Kisii | | | Kibera | | | Nakuru | | |
| --- | --- | --- | --- | --- | --- | --- | --- | --- | --- |
|  | S1 % | S2 % | *p*-value | S1 % | S2 % | *p*-value | S1 % | S2 % | *p*-value |
| Do you wear a mask? | | | | | | | | | |
| Yes | 97.0 | 99.1 | 0.091 | 98.7 | 99.6 | 0.305 | 97.4 | 98.6 | 0.359 |
| No | 3.0 | 0.9 |  | 1.5 | 0.4 |  | 2.6 | 1.4 |  |
| Frequency of mask wearing when out^a^ | | | | | | | | | |
| Always | 61.9 | 76.9 | <0.001* | 68.4 | 58.2 | 0.062 | 81.5 | 87.5 | 0.038* |
| Most of the time | 14.8 | 21.4 |  | 21.3 | 30.2 |  | 6.6 | 7.4 |  |
| Sometimes | 23.3 | 1.7 |  | 10.2 | 11.6 |  | 11.9 | 5.1 |  |
| Part of face covered when mask wearing^b^ | | | | | | | | | |
| Mouth and nose | 89.1 | 94.4 | 0.001* | 92.5 | 93.6 | 0.267 | 94.7 | 93.0 | 0.052 |
| Mouth only | 0.4 | 0.9 |  | 3.9 | 1.3 |  | 3.6 | 0.9 |  |
| Nose only | 6.5 | 0.0 |  | 0.4 | 0.4 |  | 0.9 | 0.0 |  |
| Varies | 3.9 | 4.8 |  | 3.1 | 4.7 |  | 0.9 | 6.1 |  |
| Frequency of mask re-positioning^c^ | | | | | | | | | |
| Never | 2.7 | 5.2 | <0.001* | 6.2 | 2.2 | <0.001* | 9.3 | 5.1 | <0.001* |
| Infrequently | 13.9 | 36.7 |  | 16.4 | 35.3 |  | 17.2 | 54.2 |  |
| Frequently | 53.4 | 25.3 |  | 45.8 | 39.2 |  | 41.0 | 25.0 |  |
| Very Frequently | 30.0 | 32.8 |  | 31.6 | 23.3 |  | 32.6 | 15.7 |  |
| Source of mask^d^ | | | | | | | | | |
| Gov / NGO | 10.6 | 53.7 | <0.001* | 16.1 | 25.3 | 0.029* | 7.0 | 30.6 | <0.001* |
| Shop | 87.3 | 43.2 |  | 80.5 | 69.2 |  | 84.3 | 61.2 |  |
| Internet | 0.4 | 0.0 |  | 1.3 | 3.2 |  | 2.5 | 3.6 |  |
| Self/family-made | 1.7 | 3.2 |  | 2.1 | 2.4 |  | 6.2 | 4.7 |  |
| Mask / mask material price (KES)^e^ | | | | | | | | | |
| Amount (±SD) | 51.4 ±  17.5 | 36.2 ±  22.8 | <0.001* | 49.0 ±  19.9 | 37.2 ±  25.2 | <0.001* | 53.9 ±  25.5 | 27.2 ±  22.1 | <0.001* |

**Supporting Table 2a:** Individual settlement data related to behaviours around mask use and procurement. Values are shown as percentages %, unless otherwise stated. The same sample sizes for answers to each question in this table are variable, owing to the nature of the questions asked. The superscript corresponds to the sample size of each response, shown below:

^a^Kisii Pre: 223, and Post: 229; Nairobi Pre: 225, and Post: 232; Nakuru Pre: 227, and Post: 216.

^b^Kisii Pre: 230, and Post: 231; Nairobi Pre: 228, and Post: 233; Nakuru Pre: 225, and Post: 229.

^c^Kisii Pre: 223, and Post: 229; Nairobi Pre: 225, and Post: 232; Nakuru Pre: 227, and Post: 216.

^d^Kisii Pre: 236, and Post: 285; Nairobi Pre: 236, and Post: 253; Nakuru Pre: 242, and Post: 278.

^e^Kisii Pre: 207, and Post: 123; Nairobi Pre: 192, and Post: 180; Nakuru Pre: 217, and Post: 181.

Gov: government; NGO: non-governmental organisation; SD: standard deviation; S1: Survey 1; S2: Survey 2.

*Significant at 5%

**Supporting Table 2b. Individual settlement data to behaviours around mask maintenance and training**

|  | Kisii | | | Nairobi | | | | | Nakuru | | | | |
| --- | --- | --- | --- | --- | --- | --- | --- | --- | --- | --- | --- | --- | --- |
|  | S1 % | S2 % | *p*-value | S1 % | S2 % | | *p*-value | | S1 % | S2 % | | *p*-value | |
| Are your masks reusable or not^f^ | | | | | | | | | | | | | |
| Reusable | 84.4 | 69.8 | <0.001* | 66.7 | | 50.8 | | <0.001* | 85.8 | | 41.5 | | <0.001* |
| Disposable | 8.2 | 16.3 |  | 14.5 | | 30.3 | |  | 13.0 | | 29.9 | |  |
| Sometimes both | 7.4 | 13.9 |  | 18.8 | | 18.9 | |  | 1.2 | | 28.6 | |  |
| Maintenance of reusable masks^g^ | | | | | | | | | | | | | |
| Washing | 99.5 | 100.0 | 0.341 | 98.1 | | 97.5 | | 0.693 | 98.1 | | 99.0 | | 0.427 |
| Dry cleaning | 0.0 | 0.0 |  | 1.3 | | 0.8 | |  | 0.0 | | 0.0 | |  |
| Antiseptic dipping | 0.5 | 0.0 |  | 0.0 | | 0.8 | |  | 0.5 | | 1.0 | |  |
| None of above | 0.0 | 0.0 |  | 0.6 | | 0.8 | |  | 1.4 | | 0.0 | |  |
| Washing frequency of reusable masks^g^ | | | | | | | | | | | | | |
| Daily | 63.6 | 64.2 | 0.382 | 62.2 | | 71.9 | | 0.320 | 49.3 | | 71.1 | | 0.005* |
| 2-3 days | 25.6 | 24.4 |  | 30.8 | | 20.7 | |  | 35.1 | | 25.8 | |  |
| Weekly | 6.7 | 9.7 |  | 5.1 | | 5.8 | |  | 12.3 | | 3.1 | |  |
| Fortnightly | 1.0 | 1.1 |  | 0.0 | | 0.8 | |  | 0.9 | | 0.0 | |  |
| Monthly | 3.1 | 0.6 |  | 1.3 | | 0.8 | |  | 1.9 | | 0.0 | |  |
| Never | 0.0 | 0.0 |  | 0.6 | | 0.0 | |  | 0.5 | | 0.0 | |  |
| Frequency of replacing reusable masks^h^ | | | | | | | | | | | | | |
| Daily | 30.8 | 18.2 | <0.001* | 34.0 | | 33.1 | | 0.081 | 19.4 | | 14.4 | | 0.371 |
| 2-3 days | 23.1 | 41.5 |  | 24.4 | | 17.4 | |  | 23.2 | | 20.6 | |  |
| Weekly | 14.9 | 21.6 |  | 14.1 | | 9.9 | |  | 23.7 | | 27.8 | |  |
| Fortnightly | 4.6 | 4.0 |  | 4.5 | | 3.3 | |  | 3.8 | | 9.3 | |  |
| Monthly | 15.4 | 14.8 |  | 10.9 | | 24.0 | |  | 15.6 | | 15.5 | |  |
| Never | 11.3 | 0.0 |  | 12.2 | | 12.4 | |  | 14.2 | | 12.4 | |  |
| Have you had any information or training provided on the correct use of masks^i^ | | | | | | | | | | | | | |
| Adequate info. | 10.9 | 74.5 | <0.001* | 12.3 | | 49.4 | | <0.001* | 10.7 | | 85.4 | | <0.001* |
| Some information | 50.9 | 21.6 |  | 43.9 | | 43.5 | |  | 28.8 | | 13.7 | |  |
| None | 37.8 | 3.9 |  | 43.4 | | 7.3 | |  | 59.2 | | 0.9 | |  |
| N/A | 0.4 | 0.0 |  | 0.4 | | 0.9 | |  | 1.3 | | 0.0 | |  |

**Supporting Table 2b:** Individual settlement data related to behaviours around mask maintenance and training. Values are shown as percentages %, unless otherwise stated. The same sample sizes for answers to each question in this table are variable, owing to the nature of the questions asked. Some respondents also answered more than one answer. The superscript corresponds to the sample size of each response, shown below:

^f^Kisii Pre: 231, and Post: 252; Nairobi Pre: 234, and Post: 238; Nakuru Pre: 246, and Post: 234.

^g^Kisii Pre: 195, and Post: 176; Nairobi Pre: 156, and Post: 121; Nakuru Pre: 211, and Post: 97.

^h^Kisii Pre: 195, and Post: 236; Nairobi Pre: 156, and Post: 121; Nakuru Pre: 211, and Post: 97.

^i^Kisii Pre: 230, and Post: 231; Nairobi Pre: 228, and Post: 234; Nakuru Pre: 233, and Post: 219.

S1: Survey 1; S2: Survey 2.

*Significant at 5%

**Supporting Table 3. Individual settlement data to** **attitudes to mask wearing and perceived effectiveness**

|  | Kisii | | | Nairobi | | | Nakuru | | |
| --- | --- | --- | --- | --- | --- | --- | --- | --- | --- |
|  | S1 % (n=230) | S2 %  (n=231) | *p*-value | S1 %  (n=228) | S2 %  (n=233) | *p*-value | S1 %  (n=233) | S2 %  (n=219) | *p*-value |
| Do you expect a person with chronic cough to use a face mask? | | | | | | | | | |
| Yes | 80.0 | 87.9 | 0.052 | 91.7 | 85.8 | <0.001* | 76.0 | 92.2 | <0.001* |
| No | 15.7 | 10.4 |  | 7.5 | 3.9 |  | 20.6 | 5.0 |  |
| Uncertain | 4.3 | 1.7 |  | 0.9 | 10.3 |  | 3.4 | 2.7 |  |
| Do you believe that wearing a mask is useful to prevent the spread of COVID-19? | | | | | | | | | |
| Definitely | 80.0 | 91.8 | 0.001* | 85.1 | 84.1 | 0.537 | 76.0 | 97.3 | <0.001* |
| Possibly | 17.4 | 7.4 |  | 14.0 | 13.7 |  | 20.6 | 1.8 |  |
| Do not know | 2.6 | 0.9 |  | 0.9 | 2.1 |  | 3.4 | 0.9 |  |
| Do you feel that use of mask can be harmful at a time of a respiratory pandemic? | | | | | | | | | |
| Yes | 49.6 | 44.6 | <0.001* | 60.5 | 48.1 | 0.001* | 50.6 | 19.2 | <0.001* |
| No | 40.4 | 53.2 |  | 34.2 | 37.8 |  | 41.6 | 73.5 |  |
| Uncertain | 10.0 | 2.2 |  | 5.3 | 14.2 |  | 7.7 | 7.3 |  |

**Supporting Table 3:** Individual settlement data related to attitudes to mask wearing and perceived effectiveness. Values are shown as percentages . The total sample size for each settlement in their respective pre-and-post intervention cohort is shown as (n = x) adjacent to the relevant title. S1: Survey 1; S2: Survey 2.

*Significant at 5%

**Supporting Table 4. Individual settlement data to behaviours and attitudes to COVID-19 testing**

|  | Kisii | | | Nairobi | | | Nakuru | | |
| --- | --- | --- | --- | --- | --- | --- | --- | --- | --- |
|  | S1 % | S2 % | *p*-value | S1 % | S2 % | *p*-value | S1 % | S2 % | *p*-value |
| Have you been tested for COVID-19?^j^ | | | | | | | | | |
| Yes | 5.2 | 11.7 | 0.013* | 16.2 | 30.0 | <0.001* | 8.2 | 18.3 | 0.001* |
| No | 94.8 | 88.3 |  | 83.8 | 70.0 |  | 91.8 | 81.7 |  |
| Reasons test not taken^k^ | | | | | | | | | |
| Fear quarantine | 9.2 | 5.9 | <0.001* | 9.9 | 5.5 | 0.295 | 6.1 | 7.3 | 0.148 |
| Fear positive test | 10.6 | 1.5 |  | 9.9 | 12.9 |  | 10.7 | 14.5 |  |
| Not sick | 9.2 | 7.4 |  | 25.7 | 23.3 |  | 12.1 | 17.3 |  |
| Stigma | 3.7 | 2.0 |  | 6.3 | 3.7 |  | 3.7 | 6.11 |  |
| Not available | 67.4 | 83.3 |  | 48.2 | 54.6 |  | 67.3 | 54.7 |  |
| Would you be willing to have a COVID-19 test if it were available? | | | | | | | | | |
| Yes | 76.5 | 89.6 | <0.001* | 63.6 | 60.5 | 0.495 | 67.4 | 61.2 | 0.169 |
| No | 23.5 | 10.4 |  | 36.4 | 39.5 |  | 32.6 | 38.8 |  |

**Supporting Table 4:** Individual settlement data related to behaviours and attitudes to COVID-19 testing. Values are shown as percentages %, unless otherwise stated. The sample sizes for answers to each question in this table are variable, owing to the nature of the questions asked. The superscript corresponds to the sample size of each response, shown below:

^j^Kisii Pre: 233, and Post: 231; Nairobi Pre: 228, and Post 233; Nakuru Pre: 233, and Post 219.

^k^Kisii Pre: 218, and Post: 204; Nairobi Pre: 191, and Post 163; Nakuru Pre: 214, and Post 179

S1: Survey 1; S2: Survey 2.

*Significant at 5%

**Supporting Table 5. Individual settlement data to attitudes to social distancing, and government directives**

|  | Kisii | | | Nairobi | | | Nakuru | | |
| --- | --- | --- | --- | --- | --- | --- | --- | --- | --- |
|  | S1 % | S2 % | *p*-value | S1 % | S2 % | *p*-value | S1 % | S2 % | *p*-value |
| How many metres for social distancing^L^ | | | | | | | | | |
| 1 metre | 34.8 | 9.1 | <0.001* | 43.4 | 36.1 | 0.391 | 48.9 | 39.3 | <0.001* |
| 1.5 metres | 45.2 | 83.5 |  | 39.5 | 45.9 |  | 38.2 | 42.5 |  |
| 2 metres | 18.7 | 6.9 |  | 15.4 | 39 |  | 8.6 | 18.3 |  |
| Do not know | 1.3 | 0.4 |  | 1.8 | 1.3 |  | 4.3 | 0.0 |  |
| Can people socially distance in your community^L^ | | | | | | | | | |
| Yes | 43.5 | 58.4 | 0.001* | 25.0 | 32.2 | 0.088 | 26.6 | 55.7 | <0.001* |
| No | 56.5 | 41.6 |  | 75.0 | 67.8 |  | 73.4 | 44.3 |  |
| Awareness of government COVID-19 related restrictions of travel^L^ | | | | | | | | | |
| Yes | 98.3 | 100 | 0.132 | 97.8 | 99.6 | 0.232 | 95.3 | 93.6 | 0.521 |
| No | 0.4 | 0.0 |  | 1.8 | 0.4 |  | 3.9 | 5.9 |  |
| Don’t know | 1.3 | 0.0 |  | 0.4 | 0.0 |  | 0.9 | 0.5 |  |
| Perception of government COVID-19 directives^m^ | | | | | | | | | |
| Obedience | 14.2 | 13.3 | <0.001* | 13.6 | 24.7 | 0.007* | 20.1 | 25.6 | <0.001* |
| Believe them | 64.0 | 83.1 |  | 76.7 | 70.2 |  | 60.9 | 68.8 |  |
| Mistrust | 19.2 | 3.2 |  | 8.9 | 4.7 |  | 17.9 | 4.9 |  |
| Useless | 2.5 | 0.4 |  | 0.8 | 0.4 |  | 1.1 | 0.7 |  |

**Supporting Table 5:** Individual settlement data related to attitudes to social distancing, and government directives. Values are shown as percentages % (counts). The same sample sizes for answers to each question in this table are variable, owing to the nature of the questions asked. Some respondents also answered more than one answer. The superscript corresponds to the sample size of each response, shown below:

^L^Kisii Pre: 230, and Post: 231; Nairobi Pre: 228, and Post: 233; Nakuru Pre: 233, and Post: 219

^m^Kisii Pre: 239, and Post: 248; Nairobi Pre: 236, and Post: 255; Nakuru Pre: 279, and Post: 285

S1: Survey 1; S2: Survey 2.

*Significant at 5%
