# Supplementary material for: A cross-sectional survey on the effectiveness of public health campaigns for changing knowledge, attitudes, and practices in Kenyan informal settlements during the COVID-19 pandemic
Source: PLoS One. 2023 Dec 22;18(12):e0294202. doi: 10.1371/journal.pone.0294202 (PMC10745220; doi:10.1371/journal.pone.0294202)
Supplement: Attachment — Submitted filename: Response to referees_Resubmission_Final.docx [file pone.0294202.s004.docx]

Thank you to the reviewers and editor for your comments on our manuscript. Our responses to each comment is listed below, with the original comments provided in standard text and our response underneath in *italics*.

**Editor Comments:**

1. We have double checked this and are confident that our manuscript complies with the required style.
2. We have removed this funding statement (see 3)
3. We have removed this funding statement. The correct statement should read:

*The study was funded by the Global Challenges Research Fund, QR allocations to the University of Manchester. The funders had no role in study design, data collection and analysis, preparation of the manuscript or decision to publish.*

*This is now included in the covering letter as instructed.*

4. The statement should read:

*The authors have declared that no competing interests exist.*

This is now included in the covering letter as instructed.

5. *Appendix 1 (now S1 File) is a map from OpenStreetMap*.

*Figure 1 contains map that was drawn by the authors using road data also from OpenStreetMap. These data and map images are ‘Open Data’ released under the* [*Open Data Commons Open Database License*](https://opendatacommons.org/licenses/odbl/) *as per:* [*https://www.openstreetmap.org/copyright*](https://www.openstreetmap.org/copyright)*. OpenStreetMap are properly attributed in each relevant figure (or the associated caption) and we believe that this license is compatible with the* [*CC-BY 4.0 license*](https://creativecommons.org/licenses/by/4.0/)*, so there should be no issue here.*

6. In Appendix 2 (now S2 File), we think that you are referring to our partner’s (ADRA’s) logo – we have simply removed this from the document.

**Reviewer 1 Comments:**

Reviewer #1: Title: Revise, too mouthful. The short title could be more concise and still convey the main message.

*We have shortened the title.*

Key word: Compile with the journal requirement.

*These are reproduced from the electronic submission form, rather than typed into the manuscript. We are not, therefore able to edit the format in which they are presented.*

Abstract: Provide specific numerical results related to the observed variables. Revise grammatical errors. Methodology is missing.

*We have modified the abstract to include a description of the method and to include key numerical results. The abstract now includes that it was a population-based survey using a validated bespoke questionnaire carried out at random geographical coordinates at each settlement. It also mentions that the interceding health promotion campaign and data collection was performed by trained staff. We have added more quantitative data to some of the key domains as suggested.*

**Introduction:**

a. Poor structured.

*It is difficult to know how much change is needed for this section as the second referee has stated that this was a ‘very good paper’. We have subdivided the section for greater clarity and have taken special attention to the point emphasised by you (and the editor) that we should not propagate the negative stereotypes attributed to people living under such conditions. We have been explicit in that the interchangeable use of ‘Slums’ and ‘Informal settlements’ is an error. We have made several changes to clear up potential ambiguities and hope that you would agree that the introduction is both clearer and concise as possible given the complex nature of the subject we are addressing.*

b. Limited literature review on sub-Saharan Africa, Kenya and study sites. While the introduction mentions a study in Bangladesh, it would be beneficial to discuss existing research on KAPs in informal settlements in Africa. Address the gaps in the literature that the current study aims to fill and how it expands on previous knowledge.

*We have added additional literature as requested but have highlighted the fact that these do not specifically address the question as to whether KAP can be modified through an effective public health campaign. This is where the Bangladesh study sited is important. We have also added a new more up to date reference related to KAP amongst vulnerable populations living in the Kenyan border areas in the revised manuscript, which we think is relevant. I am sure you would agree that our manuscript advances the existing literature on KAP by looking at the changes that take place over time and in response to an organised health promotion campaign. As such it addresses a gap in current literature on the subject, which we believe is now better articulated in the literature review.*

c. Consider revising some sentences to make the text clearer and more focused.

*We have made several changes throughout the text to address this point. These changes are seen in the version where the changes are tracked.*

d. Ensure that the citations follow the appropriate format according to PLOS One guidelines.

*PLOS One guidelines request Vancouver referencing style, which we have used throughout the manuscript. We have removed references to specific authors to avoid any confusion.*

e. When discussing informal settlements and slums, be cautious about the language used to avoid perpetuating negative stereotypes.

*We agree that this is of paramount importance (and note the editor’s view on this as well) – we have endeavoured to ensure that this is the case throughout. Though we could not identify any specific issues in the manuscript (and had taken our definitions from a report by UN Habitat), we have revised them further to emphasise the context of poverty and inequality.*

f. Clearly state the specific objectives of the current study in the introduction.

*Thank you. We have added new text to the concluding paragraph to the introduction making the objectives of the study clearer.* “*The study was performed to ascertain if a coordinated public health campaign, delivered by trusted local public providers, could have a positive impact on the KAPs known to be important in disease prevention”*

g. Consider including more recent statistics or relevant information on the prevalence of COVID-19 in these settlements.

*As mentioned in the manuscript, even population figures are poorly understood in these settlements, and reliable figures for COVID-19 prevalence within informal settlements are not available.*

h. Methodology: Is it quantitative, qualitative or mix method study.

*This manuscript is based on quantitative data obtained from the survey tool. Even though the survey tool had some qualitative questions, in the current manuscript only the quantitative data has been included for purposes of clarity and focus. The focus of the current manuscript is the change in KAP between the two time points and adding further qualitative data would distract the reader from this key message.*

i. Sampling should be a different topic from study design and elaborate more on sampling procedures. While it is mentioned that the population estimates were made in-house for the study, it would be beneficial to elaborate on the methodology used to estimate the population in each settlement. Mention the sample size calculation and justify it. Explain the randomization process for survey administration in more detail.

*Though the structural point is useful, we are mindful that we do not want to create too many additional level 2 headings (the ethics section, for example, comprises only a single sentence). Nevertheless, we have added a new “Sampling Strategy” section to the Materials and Methods that elaborates on the population estimates and sampling design in more detail, in addition to the existing references to other works detailing the same approach.*

j. Provide a more detailed explanation on data collection procedure that ensure the confidentiality and privacy of the participants.

*The manuscript already states that “All data was collected using anonymised codes and the study team did not collect or have access to identifiable personal data at any stage of the study.”. We believe that this achieves the requirement of assurances of privacy and confidentiality of participant information.*

k. Justification for choosing study sites not clear,

*We have elaborated on this in the Materials and Methods section.*

l. While the health promotion activities are outlined, it would be helpful to elaborate on the strategies used to evaluate the effectiveness of these interventions. Did the study team collect data on the reach and impact of the health promotion campaign?

*We are not quite clear on the precise meaning of this question, so we have tried to address it from a number of perspectives:*

*Our study was focussed on the question whether a structured campaign was effective in changing the KAPs within the relatively short time period between the two surveys (not the long-term impacts). Given the acuteness of the pandemic, this short-term change in KAP was important to document.*

*Our data show that there was a positive change in the KAP. Our promotion campaign was spread across the entire settlement (as opposed to limited to the survey points). The changes we have demonstrated (using randomly selected subjects), demonstrate a positive change across the settlement population.*

*If the query is about independent monitoring of the effectiveness of the campaign/ penetration across the settlements, the following has been added to the revised manuscript “Feedback from the QA team and the observations of the LSG group were used iteratively to ensure that the health promotion campaign reached out across the entire settlement and was not localised to any particular area based on convenience or ease of access.” In the Monitoring of Filed studies and Ethical Approval section.*

m. What are study dependent and independents variables?

*The majority of this study is comparative (before and after intervention) rather than predictive, so these terms do not apply. The exception to this would be the logistic regression section, for which the dependent variable is ‘mask wearing’ and the independent variables are listed in Table 6.*

n. Monitoring of field study should be a different topic from ethical consideration.

*As above (this comment is given twice) – we have tried to avoid introducing too many level 2 headings – in this case, ethics alone would comprise only a single sentence, so we have left it integrated within one common section. But we would however be happy to change this if the Editor would prefer.*

o. Data management is lacking.

*We have added a sentence regarding data management.*

*To be explicit here: data were collected using the Open-Source Kobo Collection software, which is part of the Kobo Toolbox suite, a standard platform for humanitarian data collection. Data are ‘end to end’ encrypted for transmission to the server. ADRA are the data controller for this dataset, and the dataset will be preserved for 7 years before deletion.*

Results:

a. The observation of potential differences in KAPs between settlements is intriguing, discuss potential reasons for these differences

*We have done this in the discussion section as per the journal’s guidelines. As stated, the study was not designed specifically to look for differences between the different types of settlements. However, during the analyses, the differences between Kibera and the other two settlements became very evident and could not go unreported. We have suggested that “The reasons could, for example, relate to differences in population density, the urban setting, quality of life, pre-existing levels of NGO activity or levels of trust with the local government/health authorities” in the revised manuscript.*

*Based on our extensive local knowledge and insights, we are aware that the Kibera settlement - due to its proximity to the capital, has higher level of activities from NGOs and other similar external organisations (universities, research groups etc.). Despite these interventions/research etc., the daily life challenges and lived experiences of the residents have largely remained unchanged. It is therefore possible that there is a greater level of suspicion/cynicism within these communities to external ‘advise’ or ‘NGO fatigue’ as mentioned in literature. We are unable to speculate any further in the current manuscript as our data is limited and this was not the primary purpose of this study. Nevertheless, the observation, in our view, is important and hence we have reported them along with a cautious and limited comment mentioned above.*

b. The authors mentioned that 39 participants were excluded from data analysis due to various reasons, such as unfeasible answers, contradictory responses, and incorrect data entries. It would be helpful to provide more specific examples of such issues to understand their impact on the data and the potential implications for the study's conclusions.

*The reasons for exclusion are as follows:*

*Survey 1 (Pre-intervention)*

- *In a typical week, how many days do you work?* *– 11 patients were removed as they answered that they worked > 7 days per week*
- *One person was removed who said they never used a mask to protect themselves against COVID-19 (when asked about frequency of use) despite saying they did wear a mask in a previous question – these answers are contradictory.*
- *Removal of n = 2 persons who explained the way they maintained their mask was ‘daily’ – this result does not describe how they maintain their mask, but the frequency they do so – in the context of the question, this result cannot be interpreted and therefore their data are removed.*
- *Removal of n = 5 people who answered that they maintain their mask through washing, but then explained that they never washed their mask when asked on the frequency. These statements are contradictory and therefore removed.*
- *4 deletions from category 8a. Time spent at home on working days as > 24 hrs.*
- *1 was removed from 8b. Time spent in others on working days as > 24 hrs*
- *1 person removed from category 8c. Time spent at work on working days as > 24 hrs*
- *2 removed from 9a. Time spent at home on non-working days as > 24 hours*
- *1 removed from 9b. Time spent In others’ homes on non-working days as > 24hrs*
- *1 removed from 10c.Time spent outside the settlement on working days as > 24 hrs*
- *1 removed from 11a.Time spent in the neighbourhood on non-working days as > 24 hrs*

*Subtotal, n=30*

*Survey 2 (Post intervention)*

- *3 people excluded who did not give consent*
- *6 people removed from 6. In a typical week, how many days do you work as > 7 days*

*Subtotal, n =9*

*The distribution of the excluded responses are as follows:*

*Survey 1: Kisii 5; Kibera 13; and Nakuru 9*

*Survey 2: Kisii 1; Kibera 3 and Nakuru 5*

*We have added some brief comments summarising the above in the revised manuscript*

c. In socio-demography why separate college, university and post-degree instead of summing it up as tertiary education.

*The questionnaire was devised through very extensive consultations with local stakeholders. It was validated for clarity with lay participants at each settlement before use. We agree that there are different ways in which educational achievement could be stratified, but we believe that maintaining the greater level of detail is valuable is worthwhile in the absence of any need to combine them.*

d. Which variable are compared against to generate the P-values indicated.

*The p values are the result of the stated tests (Chi-square, t-test etc.) - in each case they are comparisons of the results from before and after the intervention to allow us to see how things changed during this period. The specific variable being tested is listed alongside the p-value.*

Discussion:

e. Should be based on the study's findings. Expound on the discussion of the findings and addressing any limitations will strengthen the research's impact and implications for public health efforts.

*We believe that we now have done this and are happy to answer specific questions in this regard. Our discussion is entirely based on data and any extensions beyond the data is based on published literature or widely accepted wisdom/knowledge in some instance. Limitations are explained and justified. The impact and implications to future public health campaigns are presented to the best of our ability. We are pleased that Referee 2 agrees that this is a well written manuscript and the editor’s comments also seem to agree with the assessment of Referee 2.*

f. Consider providing more interpretation and discussion of the observed changes in behaviors, attitudes, and perceptions. Are there any factors that might have contributed to these changes? How do the findings align with previous research or public health campaigns? Discuss the practical implications of these changes for public health interventions.

*We believe that these are already discussed at some length. We have a dedicated heading for limitations, and the Discussion section begins by summarising the study and findings, linking back to the literature, and then reflecting on the outcomes and their implications.*

Conclusion:

g. Should be based on the study's findings. What are the key takeaways for policymakers and public health practitioners in Kenya or similar regions facing similar challenges?

*It is not immediately obvious which of our conclusions the reviewer considers not to be supported by the findings of the study. However, in response to this comment we have removed the section discussing the role of ADRA – a locally respected NGO, in the positive outcomes of the study. We agree that this section (though important) is not fully supported by our data.*

*We agree that the take home messages are important and need to be articulated better, and we have edited the manuscript accordingly.*

h. Language and Clarity: The manuscript's language should be revised for clarity and precision. Some sentences appear convoluted and may benefit from rephrasing. Additionally, ensure consistent use of terminology throughout the manuscript.

*We have attempted to simplify the language further throughout the manuscript to appeal to a wide readership and for greater clarity and precision. We are however grateful that this view was not shared by the second referee who has stated that this was a ‘very good’ manuscript.*

**Reviewer 2:**

a. Very good paper.

*Thank you for this comment*.

b. Statistical analysis: please clarify with analysis package was used, is it SPSS or STATA? the paper mentions both as(SPPS or STATA)

*SPSS was used. The erroneous reference to STATA has now been removed.*

c. The intervention was community education through various means including radio, use of CHVs and health providers, however, given the abundance of information at that time, the authors should mention the possible role/influence/limitations of social media on the outcomes.

*The following section has been added to the manuscript: “The public health campaigns conducted by the regular government public health officials – utilising similar methodologies and other social media platforms continued unimpeded by the focussed campaign carried out as part of the present study*” in the section ‘Health Promotion and the conduct of field work’. The discussion section also alludes to the dose effect our campaign may have had in this context.

d. Methods: Its mentioned, the population was stratified, and purposively selected, from outdoor participants, how did the study team ensure that the was no bias with only those interested in the study taking part? how were the two participants identified to reduce bias?

*The sampling strategy section is now rewritten with more details. The location was selected randomly using a GIS based approach and at each sampling location the first two subjects agreeing to take part in the study were recruited. It is possible that this approach carries the risk of selecting only those subjects who were willing to engage and thereby introduce some bias in our sampling process, though it is impossible to quantify the magnitude of this risk and how it may have influenced our findings. This is a risk which is inherent to across all population-based surveys and hard to mitigate against in these settings. This is now discussed in the manuscript under section on the limitations of the study.*

e. Details on Mask distribution: Given the population in the informal settlements, how were the 60,000 masks distributed? How does that relate to the 720 respondents’ access to the masks? were the 720 respondents targeted to receive the masks? being repeated cross sectional surveys, the respondents at the at baseline and endline were different, how does that relate to the access to the distributed masks?

*We agree that this is an important point and should have been explained better. The following statement has been added in the revised manuscript:*

*“The distribution of sanitisers and masks were carried out across the community as part of the campaign and was independent of the two surveys to ensure that the survey responses were independent and not influenced by the incentives of free masks, sanitisers or information leaflets”.*

*We hope this addresses the above point raised by the referee.*

f. Was consenting part of the process? please indicate under ethics.

*Consent was included. It was previously only described in the results section (under exclusion criteria) – we have now included this in the ethics section as well.*
